# Clinical practice of diabetic pregnancy screening in Asia-Pacific Countries: a survey review

**DOI:** 10.1007/s00592-019-01331-8

**Published:** 2019-04-06

**Authors:** Ling-Jun Li, Qi Yu, IPRAMHO-INTERNATIONAL Study Group, Alexis Shub, Chen Weiqing, Jim Zhang Jun, Mamoru Tanaka, Krishna Kuma, G. Muniswaran, Swe Swe Myint, Yin Yin Soe, Valerie T. Guinto, Tony Tan, Serene Thain, Kok Hian Tan, Claudia Chi, Lay Kok Tan, Shahul Hameed Mohamed Siraj, Ounjai Kor-ananatakul, Dittakarn Boriboonhirunsarn, Kok Hian Tan

**Affiliations:** 1 Division of O&G, KK Women’s and Children’s Hospital, 100 Bukit Timah Road, Singapore, 229899 Singapore; 2 OBGYN ACP, Duke-NUS Medical School, Singapore, Singapore; 3 Duke Medical School, Duke University, Durham, NC USA

**Keywords:** Gestational diabetes mellitus, Oral glucose tolerance test, Metabolic health, Asia-Pacific countries, Screening, International Association of the Diabetes and Pregnancy Study Groups (IADPSG)

Gestational diabetes (GDM) is the development of glucose intolerance during pregnancy in women without pre-gestational diabetes [1]. Although the International Association of the Diabetes and Pregnancy Study Groups (IADPSG) guidelines for GDM diagnosis has been adopted worldwide [2], little is known about the screening and diagnosis of GDM in most Asia-Pacific countries such as Thailand, Myanmar, Malaysia and Philippines. Therefore, it is necessary to understand the current GDM risk and/or universal screening approaches in Asian countries, since GDM is becoming increasingly prevalent in Asian countries in the recent decades [3]. In this manuscript, we conducted a survey regarding GDM screening, treatment and follow-up strategy among nine participating Asia-Pacific countries during the first Integrated Platform for Research in Advancing Metabolic Health Outcomes of Women and Children (IPRAMHO) Asia-Pacific workshop which was held in Singapore. We aimed to summarize and also provided some insights of GDM screening practices in more under-reported Asia-Pacific regions/countries.

A total of 12 surveys were sent out to clinician and academic attendees from nine participating Asia-Pacific countries, namely Singapore, Philippines, Australia, Myanmar, Japan, Malaysia, Thailand, China, and Sri Lanka. The response rate was 100%. The survey respondents were selected from all country representative participants of the first IPRAMHO Asia-Pacific workshop, which involved 60% of ASEAN countries (6 out of 10). The surveys comprised eight sections which collected information on respondent demographics, current GDM policy, screening for pre-existing diabetes, screening for GDM before and after 24 weeks of gestation, GDM policy for delivery, and GDM policy for follow-up after delivery (Supplementary Annex 1).

We summarized the detailed information of each survey respondent and affiliated hospital in Table 1. Responders were almost equally divided between clinicians and academic professors. The number of deliveries per year at each hospital ranged from less than 1000 (1/12, 8.3%) to greater than 10,000 (4/12, 33%). All surveyed hospitals had a national policy or regional guideline regarding pre-existing diabetes and GDM screening (Supplementary Fig. 1).

**Table 1:** Survey respondents’ characteristics and representative hospitals and countries

Respondent number	Country	City	Hospital	Clinician	Academic researcher	# of obstetricians	# of midwives	# of deliveries/year
1	China	Shanghai	Xinhua Hospital		√	10	0	3600
2	China	Guangzhou	First Affiliated Hospital of Sun Yat-sen University	√		35	40	5400
3	Thailand	Bangkok	Siriraj Hospital		√	60	80	8000
4	Thailand	Hat Yai	Songklanagarind Hospital	√		30	30	3600
5	Malaysia	Kuala Lumpur	Kuala Lumpur General Hospital	√		12	160	12,000
6	Malaysia	Negeri Sembilan	Tuanku Jaafar Seremban	√		8	N/A	11,000
7	Japan	Tokyo	Keio University Hospital	√		40	25	600
8	Myanmar	Yangon	Central Women’s Hospital		√	54	144	14,659
9	Myanmar	Yangon	Central Women’s Hospital		√	54	144	14,659
10	Australia	Melbourne	Mercy Hospital for Women	√		30	100	5800
11	Philippine	Manila	University of the Philippines, Philippine General Hospital	√		60	20	5000
12	Singapore	Singapore	KK Women’s and Children’s Hospital	√		40	20	12,000
13	Sri Lanka	Batticaloa	Teaching Hospital Batticaloa	√		4	30	6000

Regarding the screening for pre-existing diabetes, the majority of survey respondents (7/12, 58.3%) had a policy of using a risk profile assessment, with commonly assessed factors including family history of diabetes (9/12, 75%), previous onset of GDM (9/12, 75%), pre-pregnancy BMI (8/12, 66.7%) and others (i.e., prior macrosomia) (Supplementary Fig. 2). Countries that had universal screening (100%) for pre-existing diabetes were Malaysia, China (Shanghai), and Japan. For screening of GDM before 24 weeks of pregnancy, all survey respondents were analyzed except for Sri Lanka and China (Guangzhou) (10/12, 83.3%). All hospitals screened for GDM used different guidelines (Supplementary Fig. 3) and most of them (8/10, 80%) were screened based on risk factors such as: previous onset of GDM (8/8, 100%), family history of diabetes (7/8, 87.5%), and pre-pregnancy BMI (5/8, 62.5%). All hospitals screened for GDM beyond 24 weeks of pregnancy (Universal screening vs. risk screening: 66.7% vs. 33.3%). The estimated percentage of pregnant women who receive screening ranged from 69 to 100% and most were screened from 24 weeks onwards (8/12, 66.7%). The screening tests varied, but the most commonly used was the one-step IADPSG guidelines (6/12, 50%) (Table 2).

**Table 2:** Current screening tests used for GDM screening after 24 weeks of pregnancy

OGTT type	Criteria	Country
One step with 75 g 2 h	IADPSG: One abnormal value of fasting ≥ 5.1 mmol/L, 1-h ≥ 10 mmol/L or 2-h ≥ 8.5 mmol/L	Guangzhou, China Shanghai, China Tokyo, Japan Melbourne, Australia Singapore Hat Yai, Thailand
	WHO 2006: One abnormal value of fasting ≥ 7 mmol/L and/or 2-h ≥ 11.1 mmol/L	Yangon, Myanmar
	NICE: One abnormal value of fasting ≥ 5.6 mmol/L and/or 2-h ≥ 7.8 mmol/L	Negeri Sembilan, Malaysia
	Nation-specific: Fasting ≥ 5.1 mmol/L and 2 h ≥ 7.8 mmol/L	Kuala Lumper, Malaysia
	POGS criteria: fasting ≥ 5.1 mmol/L, 1-h ≥ 10 mmol/L or 2-h ≥ 7.8 mmol/L	Manila, Philippine
	Nation-specific: : FBS criteria: One abnormal value of fasting ≥ 100 mg/dL, 1-h ≥ 180 mg/dL or 2-h ≥ 140 mg/dL	Batticaloa, Sri Lanka
Two step with 50 g 1 h followed by 100 g 3 h	ACOG: Two abnormal values of fasting ≥ 5.3 mmol/L, 1-h ≥ 10.0 mmol/L, 2-h ≥ 8.6 mmol/L or 3-h ≥ 7.8 mmol/L	Bangkok, Thailand Hat Yai, Thailand

*IADPSG* International Association of Diabetes and Pregnancy Groups, *WHO* World Health Organization, *NICE* National Institute for Clinical Excellence, *POGS* Philippine Obstetrical and Gynecological Society, *ACOG* Obstetricians and Gynecologists

All surveyed hospitals had a protocol for GDM treatment at delivery except for Sri Lanka. Most surveyed centers (9/12, 75%) had a protocol for long-term follow-up of women with GDM especially concerning the risk of development of type 2 diabetes (T2D). The majority of centers surveyed performed oral glucose tolerance testing (OGTT) between 6 and 12 weeks postpartum (9/12, 75%) and provided advice on postnatal care (Supplementary Table 1).

All 12 hospital centers that participated in this survey had national policies or guidelines regarding GDM screening. One-step IADPSG criteria was the most common guideline used. However, the methods of screening for pre-existing diabetes and postpartum GDM follow-up varied greatly.

Generally, women from 24 weeks of gestation onwards are about twice as likely to be diagnosed with GDM using universal screening than screening based on risk factors [4]. In our survey review, hospitals were twice as likely to employ universal screening as risk factor-based screening. A recent Finnish cohort—investigating the accuracy of different prediction models in women at risk of GDM—found that marked heterogeneity of GDM challenged the development of risk scores for detection of GDM. In other words, the traditional risk factor-based GDM screening is flawed and insufficient for GDM diagnosis [5]. Therefore, more research is needed on comparing the effect of universal vs. risk factor-based screening for GDM in Asian ethnic groups on maternal and fetal outcomes.

Even though one-step IADPSG criteria was the most commonly adopted, only half of the surveyed hospitals currently use this guideline. Delay or hesitation in adopting this evidence-based criteria might be due to lack of evidence in a Southeast Asian population and limited government resources. It is also challenging to compare prevalence among countries or regions with different diagnostic guidelines, especially in Asian countries where GDM prevalence is known to be higher than other races/ethnicities. Therefore, further research on maternal and fetal outcomes related to IADPSG guidelines diagnosed GDM in Asian countries is warranted to provide more clinical evidence that IADPSG guidelines are beneficial and generalizable to diagnosing pregnant women with GDM in the Asia-Pacific regions.

The strengths of this review include comprehensive information regarding glucose screening approaches in pregnancy, especially among countries whose clinical practice was still unknown, and an excellent response rate of 100%. However, our review is not without limitations. First, we only recruited nine countries in this survey, and the small sample might have selectively biased our results. Second, our findings potentially lack generalizability for each country as only one tertiary hospital was surveyed. Third, since only clinicians or researchers participated in the survey, there might be information bias regarding the glucose screening approaches in general.

In summary, we found that the IADPSG guideline is the most widely adopted criteria in Asia-Pacific countries for GDM screening. Countries vary widely in terms of pre-existing diabetes screening and postpartum follow-up for GDM. Studying the associations of IADPSG and other guidelines with maternal and fetal outcomes will provide greater clinical utility and guide decisions on which guidelines are most appropriate for each country.

## Electronic supplementary material

Below is the link to the electronic supplementary material.

Supplementary material 1 (JPG 84 KB)

Supplementary material 2 (JPG 66 KB)

Supplementary material 3 (JPG 111 KB)

Supplementary material 4 (DOCX 121 KB)

Supplementary material 5 (DOCX 18 KB)

## Abbreviations

GDM: Gestational diabetes
IADPSG: International Association of Diabetes and Pregnancy Groups
IPRAMHO: Integrated Platform for Research in Advancing Metabolic Health Outcomes of Women and Children
T2D: Type 2 diabetes
OGTT: Oral glucose tolerance test
