# Supplementary figures and images for: Clinical practice of diabetic pregnancy screening in Asia-Pacific Countries: a survey review
Source: Acta Diabetol. 2019 Apr 6;56(7):815–7. doi: 10.1007/s00592-019-01331-8 (PMC6557867; doi:10.1007/s00592-019-01331-8)

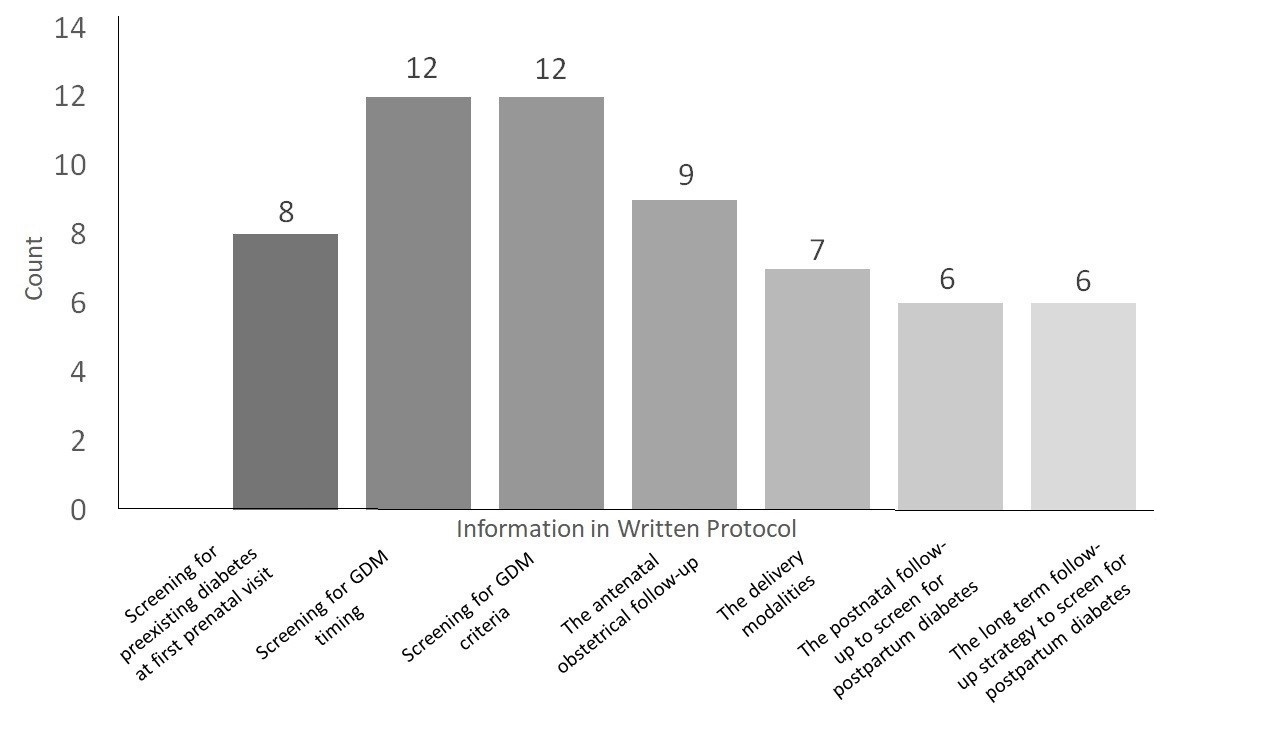

Supplement: Supplementary file 1 — Supplementary material 1 (JPG 84 KB) [file 592_2019_1331_MOESM1_ESM.jpg]

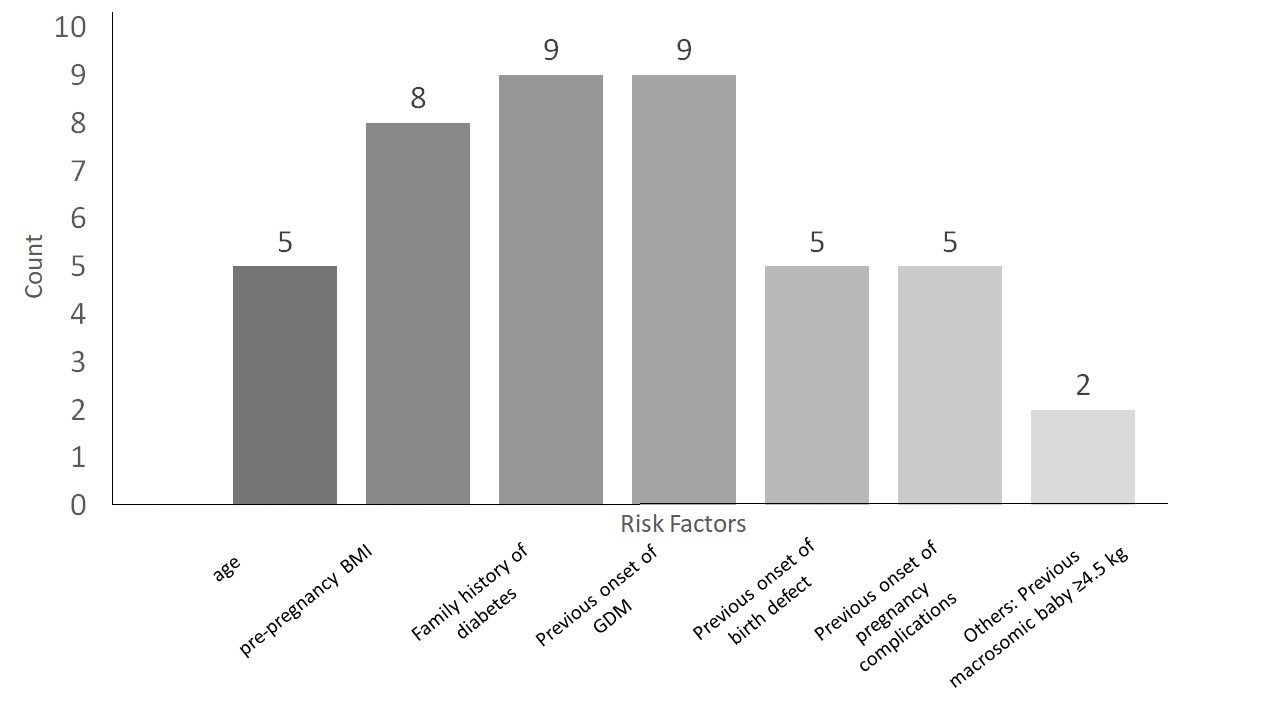

Supplement: Supplementary file 2 — Supplementary material 2 (JPG 66 KB) [file 592_2019_1331_MOESM2_ESM.jpg]

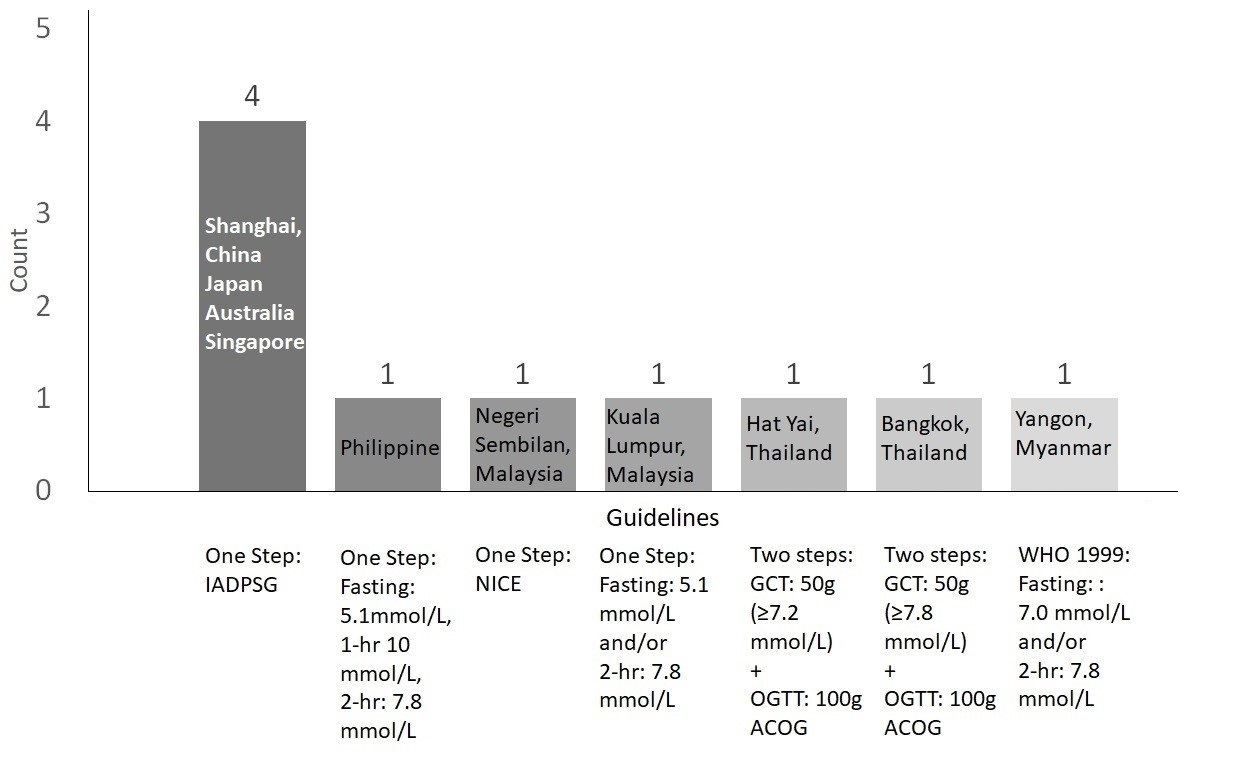

Supplement: Supplementary file 3 — Supplementary material 3 (JPG 111 KB) [file 592_2019_1331_MOESM3_ESM.jpg]
