# Supplementary material for: Clinical practice of diabetic pregnancy screening in Asia-Pacific Countries: a survey review
Source: Acta Diabetol. 2019 Apr 6;56(7):815–7. doi: 10.1007/s00592-019-01331-8 (PMC6557867; doi:10.1007/s00592-019-01331-8)
Supplement: Supplementary file 4 — Supplementary material 4 (DOCX 121 KB) [file 592_2019_1331_MOESM4_ESM.docx]

**
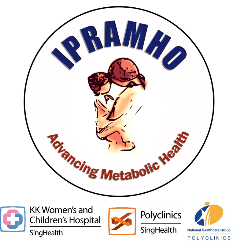
**
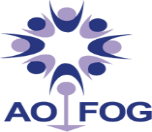
 **Singapore Diabetes in**

**Pregnancy Conference**

**& IPRAMHO-Asia Meeting 2018**

**Consensus Workshop Survey**

***Please tick the most applicable answer(s) to each question, based on your local practice.***

1. **Respondent information:**

Country:

Hospital:

Designation:

Age:  20-29 years  30-39 years  40-49 years  50-59 years  60-69 years  ≥70 years

1. **Demographics:**
2. Center:

University hospital

Non-university training hospital

Community based hospital

Other, please specify:

1. The number of obstetricians working in the hospital:

Approximately__________

1. The number of mid-wives working in the hospital:

Approximately__________

1. The average number of deliveries per year in your center:

Approximately__________

1. Does your center have a database with the number of GDM women registered/identified by OGTT values?

Yes  No  Not sure

1. What percentage of women have on average GDM in your center?

Approximately__________ %  Not sure

1. **Policy:**
2. Is there a national policy, regional guideline or diabetes association policy which your center/hospital can refer to in preexisting diabetes and GDM screening?

Yes what policies?____________  No  Not sure

1. Does you center have a written protocol concerning the policy on GDM?

Yes  No  Not sure

1. The protocol contains information on: (*More than one answer can be chosen, if applicable)*

Screening for pregestational diabetes at first prenatal visit

Screening for GDM timing

Screening for GDM criteria

The antenatal obstetrical follow up

The delivery modalities

☐ The postnatal follow-up to screen for diabetes postpartum

The long term follow-up strategy to screen for diabetes after delivery

1. Which of the following physicians and/or paramedics were involved in the development of the protocol? (*More than one answer can be chosen, if applicable*.)

Obstetrician  Endocrinologist  Pediatrician  Family physician

Midwife  Diabetes specialist nurse  Dietician

Others, please specify:

1. **Screening at first prenatal visit for *preexisting diabetes*:**
2. What is the policy in your center concerning screening for pregestational diabetes at first prenatal visit? (*More than one answer can be chosen, if applicable*.)

Leave it to the physician to decide

Assessment of the risk profile and if necessary followed by further testing

Measurement of a fasting plasma glucose

Measurement of Hba1c

Measurement of a random glycaemie

Measurement of glucosuria

Others, please specify:

1. When you use an assessment of the risk profile, which risk factors do you use? (*More than one answer can be chosen, if applicable*.)

Pregnant woman’s age

Pregnant women’s pre-pregnancy body mass index (BMI)

Pregnancy woman’s family history of diabetes

Pregnancy woman’s previous onset of GDM

Pregnancy woman’s previous onset of birth defect

Pregnancy woman’s previous onset of pregnancy complications

Others, please specify:

1. When you screen in early pregnancy, what is the estimated percentage of pregnant women who receive screening?

Approximately________________ %  Not sure

1. What is the estimated percentage of women who attend a preconception clinic?

Approximately________________ %  Not sure

1. **Screening for gestational diabetes *before 24 weeks of pregnancy***
2. Do you screen for GDM before 24 weeks of pregnancy?  Yes  No

- If you screen, what is the estimated percentage of women who receive screening before 24 weeks of pregnancy?

Approximately________________ %  Not sure

- If you screen for GDM before 24 weeks of pregnancy, is this based on:

Risk factors  Universal

1. If you screen based on risk factors, which risk factors do you use? (*More than one answer can be chosen, if applicable*.)

Pregnant woman’s age

Pregnant women’s pre-pregnancy body mass index (BMI)

Pregnancy woman’s family history of diabetes

Pregnancy woman’s previous onset of GDM

Others, please specify:

1. Which of the following screening tests do you use? (*More than one answer can be chosen, if applicable*.)

Measurement of a fasting plasma glucose

Measurement of Hba1c

Measurement of a random glycaemie

Measurement of glucosuria

Glucose challenge test:

50g with a cutoff of 7.2mmol/L

50g with a cutoff of 7.8mmol/L

Others, please specify:

Immediately an OGTT:

2-h 75g

3-h 100g

1. Which diagnostic criteria do you use with a 75g OGTT?

IADPSG: 2 abnormal values of fasting ≥ 5.1mmol/L, 1-h ≥ 10mmol/L or 2-h ≥

8.5mmol/L

WHO 1999: 1 abnormal value of fasting ≥ 7.0mmol/L or 2-h ≥ 7.8mmol/L

NICE: 1 abnormal value of fasting≥5.6mmol/L and/or 2-hour≥7.8mmol/L

others, please specify____________________

1. Which diagnostic criteria do you use with a 100g OGTT?

ACOG: 2 abnormal values of fasting ≥ 5.3mmol/L, 1-hour≥10.0mmol/L, 2-

hour≥8.6mmol/L or 3-hour≥7.8mmol/L

NDDG: 2 abnormal values of fasting ≥ 5.8mmol/L, 1-hour≥ 10.6mmol/L, 2-hour≥

9.2mmol/L or 3-hour≥ 8.0mmol/L

CDA: 2 abnormal values of fasting ≥ 5.3mmol/L, 1-hour≥ 10.6mmol/L and 2-hour≥

8.9mmol/L

☐ others, please specify____________________

1. **Screening for gestational diabetes *≥ 24 weeks of pregnancy* (usually 24-28 weeks gestation):**
2. Do you screen for GDM ≥24 weeks of pregnancy?  Yes  No

- If yes, what is the estimated percentage of women who receive screening ≥24 weeks of pregnancy?

Approximately________________ % %  Not sure

- If yes, is it based on?

Risk factors  Universal

1. If you screen based on risk factors, which risk factors do you use? (*More than one answer can be chosen, if applicable*.)

Pregnant woman’s age

Pregnant women’s pre-pregnancy body mass index (BMI)

Pregnancy woman’s family history of diabetes

Pregnancy woman’s previous onset of GDM

Others, please specify:

1. From how many weeks till how many weeks do you generally screen for GDM?

22 weeks onwards

24 weeks onwards

26 weeks onwards

28 weeks onwards

1. Which of the following screening tests do you use?

Measurement of a fasting plasma glucose

Measurement of Hba1c

Measurement of a random glycaemie

Measurement of glucosuria

Glucose challenge test:

50g with a cutoff of 7.2mmol/L

50g with a cutoff of 7.8mmol/L

Others, please specify:

Immediately an OGTT:

2-h 75g

3-h 100g

1. Which diagnostic criteria do you use with a 75g OGTT?

IADPSG: 2 abnormal values of fasting ≥ 5.1mmol/L, 1-h ≥ 10mmol/L or 2-h ≥

8.5mmol/L

WHO 1999: 1 abnormal value of fasting ≥ 7.0mmol/L or 2-h ≥ 7.8mmol/L

NICE: 1 abnormal value of fasting≥5.6mmol/L and/or 2-hour≥7.8mmol/L

☐ others, please specify____________________

☐ Not using this

1. Which diagnostic criteria do you use with a 100g OGTT?

ACOG: 2 abnormal values of fasting ≥ 5.3mmol/L, 1-hour≥10.0mmol/L, 2-

hour≥8.6mmol/L or 3-hour≥7.8mmol/L

NDDG: 2 abnormal values of fasting ≥ 5.8mmol/L, 1-hour≥ 10.6mmol/L, 2-hour≥

9.2mmol/L or 3-hour≥ 8.0mmol/L

CDA: 2 abnormal values of fasting ≥ 5.3mmol/L, 1-hour≥ 10.6mmol/L and 2-hour≥

8.9mmol/L

☐ others what____________________

☐ Not using this

1. What treatments are available at your center for women diagnosed with GDM during pregnancy? (*More than one answer can be chosen, if applicable*.)

Refer all women to a dietitian

Advise them regular exercise to improve glycemic control

Educate on capillary blood glucose monitoring to maintaining glucose levels (fasting

<5.5 mmol/L, 2-hour<6.6mmol/L)

Assess the best treatment option which can include insulin and/or metformin

All of the above

None of the above, please indicate:

1. Has your hospital ever used other guideline before it changed to IADPSG?

Yes.

If so, what guideline was used before?

If so, when was it changed to IADPSG?

Not applicable

1. When did your hospital change to routine screening for GDM

If yes, please specify:

Not applicable

1. **The policy concerning gestational diabetes *at delivery*:**
2. Does your center have a protocol for GDM treatment at delivery concerning: (*More than one answer can be chosen, if applicable*.)

Monitoring blood glucose

The need of an insulin sliding scale

The need for an induction

The need for a caesarean section

Neonatal care on the monitoring of blood glucose in newborns

The need for admission on the neonatal intensive care unit

Others, please indicate:

1. **The long term policy in GDM patients *after delivery* concerning the risk to develop type 2 diabetes**
2. Do you have a protocol concerning the long term follow up of the risk of women with previous GDM to develop type 2 diabetes after the delivery?

Yes  No  Not sure

1. The follow up strategy includes the following:

Monitoring blood glucose in hospital after the delivery

Self-monitoring of blood glucose at home

A 75g OGTT only in insulin treated women

An universal 75g OGTT postpartum

Fasting plasma glucose

HbA1c

Random glycaemia

1. When an OGTT is performed postpartum, this is:

<6 weeks postpartum

Between 6-12 weeks postpartum

> 12 weeks postpartum

Others, please specify:

1. If screening is done and OGTT is not performed postpartum, what is the screening tool and criteria:

Others, please specify:

1. The advice given to women with previous GDM, includes:

Diet and weight control

Physical activity

The frequency and manner of sceening for type 2 diabetes after delivery

The need for preconception control when planning a new pregnancy

Advice on the preferred choice of contraceptives

Others, please specify:

1. What is the regular screening frequency in GDM patients in your center?

Yearly

2-yearly

3-yearly

Not sure

1. If follow-up is done, what is the screening tool and criteria, if any please spedify:
