# Supplementary material for: Clinical practice of diabetic pregnancy screening in Asia-Pacific Countries: a survey review
Source: Acta Diabetol. 2019 Apr 6;56(7):815–7. doi: 10.1007/s00592-019-01331-8 (PMC6557867; doi:10.1007/s00592-019-01331-8)
Supplement: Supplementary file 5 — Supplementary material 5 (DOCX 18 KB) [file 592_2019_1331_MOESM5_ESM.docx]

Supplementary Table 1. Postnatal screening guidelines, screening frequency, and follow-up strategy for women with GDM

| Country | Postnatal Screening Protocol | 75 g OGTT performed Postpartum | Timing of OGTT | Screening frequency | Diet and weight control | Physical activity | Frequency and manner of screening for type 2 diabetes after delivery | Need for preconception control when planning a new pregnancy | Advice on the preferred choice of contraceptives | Other |
| --- | --- | --- | --- | --- | --- | --- | --- | --- | --- | --- |
| Shanghai, China | X | N/A | N/A | Not sure | √ | √ | √ |  |  |  |
| Guangzhou, China | √ | √ | 6-12 weeks | 3-yearly | √ | √ | √ | √ |  |  |
| Negeri Sembilan, Malaysia | X | Yes | 6-12 weeks | None |  |  |  |  |  | Other: Screen postpartum and during next pregnancy |
| Kuala Lumper, Malaysia | X | N/A | 6-12 weeks | N/A |  |  |  |  | √ |  |
| Tokyo, Japan | √ | √ | 6-12 weeks | Not sure | √ | √ | √ | √ |  |  |
| Manila, Philippines | √ | √ | >6 weeks | Yearly | √ | √ | √ | √ | √ |  |
| Batticaloa, Sri Lanka | √ | X (FPG) | N/A | Not sure | √ | √ | √ | √ | √ |  |
| Singapore | √ | √ | 6-12 weeks | Not sure | √ | √ | √ |  |  |  |
| Thailand (Bangkok and Hat Yai) | √ | √ | 6-12 weeks | Yearly | √ | √ | √ | √ | √ |  |
| Yangon, Myanmar | √ | X (FPG) | 6-12 weeks | Yearly | √ | √ | √ | √ | √ |  |
| Melbourne, Australia | √ | √ | 6-12 weeks | 2-yearly | √ | √ | √ |  |  |  |
